# Exceptional sustained long-term complete response to Tepotinib in a *MET*-amplified advanced intrahepatic biliary tract cancer failing Durvalumab plus Cisplatin and Gemcitabine

**DOI:** 10.1093/oncolo/oyae265

**Published:** 2024-10-02

**Authors:** Andreas Reichinger, Leo Essl, Paul Kerschner, Jonathan Burghofer, Gerald Webersinke, Holger Rumpold, Bernhard Doleschal

**Affiliations:** Department of Internal Medicine I for Hematology with Stem Cell Transplantation, Hemostaseology, and Medical Oncology, Ordensklinikum Linz - Barmherzige Schwestern Site, Seilerstaette 4, 4020 Linz,Austria; Medical Faculty, Johannes Kepler University Linz, Altenberger Strasse 69, 4040 Linz, Austria; Medical Faculty, Johannes Kepler University Linz, Altenberger Strasse 69, 4040 Linz, Austria; Medical Faculty, Johannes Kepler University Linz, Altenberger Strasse 69, 4040 Linz, Austria; Laboratory for Molecular Genetic Diagnostics, Ordensklinikum Linz - Barmherzige Schwestern Site, Seilerstaette 4, 4020 Linz, Austria; Laboratory for Molecular Genetic Diagnostics, Ordensklinikum Linz - Barmherzige Schwestern Site, Seilerstaette 4, 4020 Linz, Austria; Department of Internal Medicine I for Hematology with Stem Cell Transplantation, Hemostaseology, and Medical Oncology, Ordensklinikum Linz - Barmherzige Schwestern Site, Seilerstaette 4, 4020 Linz,Austria; Medical Faculty, Johannes Kepler University Linz, Altenberger Strasse 69, 4040 Linz, Austria; Department of Internal Medicine I for Hematology with Stem Cell Transplantation, Hemostaseology, and Medical Oncology, Ordensklinikum Linz - Barmherzige Schwestern Site, Seilerstaette 4, 4020 Linz,Austria; Medical Faculty, Johannes Kepler University Linz, Altenberger Strasse 69, 4040 Linz, Austria

**Keywords:** *MET* amplification, *TP53* mutation, Tepotinib, biliary tract cancer, Durvalumab

## Abstract

**Background:**

Biliary tract cancers (BTCs) are a diverse group of malignancies with varied genetic backgrounds. The prevalence of intrahepatic cholangiocarcinoma (iCC) is increasing, particularly in Western countries. Despite advancements in treatments, the prognosis for BTC remains poor. Recent molecular profiling has revealed that up to 40% of iCC cases have targetable genetic alterations. *MET* amplification, although rare, presents a significant target for therapy.

**Case Presentation:**

A 25-year-old female with a history of ulcerative colitis presented with shoulder pain and a positron emission tomography–computed tomography (PET–CT) scan revealed an enlarged liver and multiple metastases. Histopathological analysis diagnosed poorly differentiated adenocarcinoma. First-line therapy with Cisplatin, Gemcitabine, and Durvalumab resulted in disease progression. Molecular profiling identified a *TP53* mutation and *MET* amplification. Based on these findings, Tepotinib was initiated. Tepotinib treatment led to a significant reduction in tumor size and normalization of CA 19-9 levels within 2 months, achieving a complete metabolic remission lasting up to 17 months. The treatment was well tolerated with minimal side effects.

**Discussion:**

*MET*-amplified BTCs are exceedingly rare, and evidence for targeted treatment is limited. This case demonstrates the efficacy of Tepotinib in a young patient with *MET*-amplified iCC, showing a long-term response and suggesting a potential new standard treatment option for this molecularly defined entity. This case also highlights the aggressive nature of *MET*-amplified tumors and the need for targeted second-line therapies.

**Conclusion:**

Tepotinib showed remarkable efficacy in treating *MET*-amplified intrahepatic cholangiocarcinoma, underscoring the importance of molecular profiling in BTCs and suggesting a potential new therapeutic approach for this rare cancer subtype.

## Teaser

Recent molecular profiling has revealed that up to 40% of intrahepatic cholangiocarcinoma cases have targetable genetic alterations. *MET* amplification, although rare, presents a significant target for therapy.

**Key Points**

• *MET* amplification, although rare in BTCs (2%-4%), is more commonly found in early-onset BTC.
• *MET* amplification drives aggressive tumor behavior and may contribute to resistance against the novel first-line standard of chemotherapy + checkpoint inhibitor, underscoring the need for targeted therapies in the second-line setting.
• Tepotinib is an effective and well-tolerated treatment for *MET*-amplified BTC, addressing a critical unmet need in managing this challenging and molecularly defined cancer subtype.

## Introduction

Biliary tract cancers (BTCs) represent a heterogeneous group of malignancies with varied anatomical distribution and genetic backgrounds.^1^ In Western countries, the prevalence of intrahepatic cholangiocarcinoma (iCC) has been increasing, a trend attributed not only to lifestyle factors but also to advancements in diagnostic techniques.^2^ Despite recent progress in first-line treatments, such as the incorporation of checkpoint inhibitors like Durvalumab or Pembrolizumab alongside traditional chemotherapy, the prognosis for BTC remains poor, with overall survival rates comparable to those of metastatic pancreatic carcinoma.^3,4^

The failure of first-line therapy leaves limited options, with only 1 phase 3 trial providing evidence for subsequent treatments. However, the introduction of broad molecular profiling in clinical practice has revealed that up to 40% of iCC cases harbor genetic alterations that are targetable, a strategy successfully employed in other tumor types. Consequently, BTC is often termed the “lung cancer of the gastrointestinal tract,” necessitating mandatory genetic profiling similar to the treatment protocols for non–small cell lung cancer.^5-7^

Certain genetic alterations in BTC are more common and supported by robust evidence for targeted treatments, while others are rare, and often documented in case reports or small-scale studies. In this context, we present a case of *MET*-amplified intrahepatic BTC that responded to molecularly informed therapy with Tepotinib, a MET inhibitor routinely used in the treatment of *MET*-altered lung cancer.^8^

## Case presentation

A 25-year-old female presented in November 2022 with bilateral shoulder pain persisting for 4 weeks and pain radiating from the spine to the upper abdomen. A positron emission tomography–computed tomography (PET–CT) scan revealed a massively enlarged liver extending to the spleen, a suspicious mass in the left liver lobe, and additional diffusely distributed lesions in the right liver lobe. Additionally, multiple pathological lymph nodes were identified in the mediastinal, paraesophageal, and retroperitoneal regions, alongside diffuse bone metastasis. The patient’s medical history included ulcerative colitis and prior idiopathic pancreatitis, with an unremarkable family history and an excellent ECOG performance status.

Histopathological analysis of an ultrasound-guided biopsy from the left liver lesion confirmed a diagnosis of poorly differentiated adenocarcinoma, classified as pancreatobiliary type based on immunomorphological markers. The immunohistochemical profile was positive for CK7 and CK19 but negative for CDX, GATA3, and TTF-1. The tumor was also PD-L1 negative and microsatellite stable (MSS), leading to the diagnosis of mass-forming intrahepatic cholangiocarcinoma.

### Treatment and disease progression

**Figure 1.**
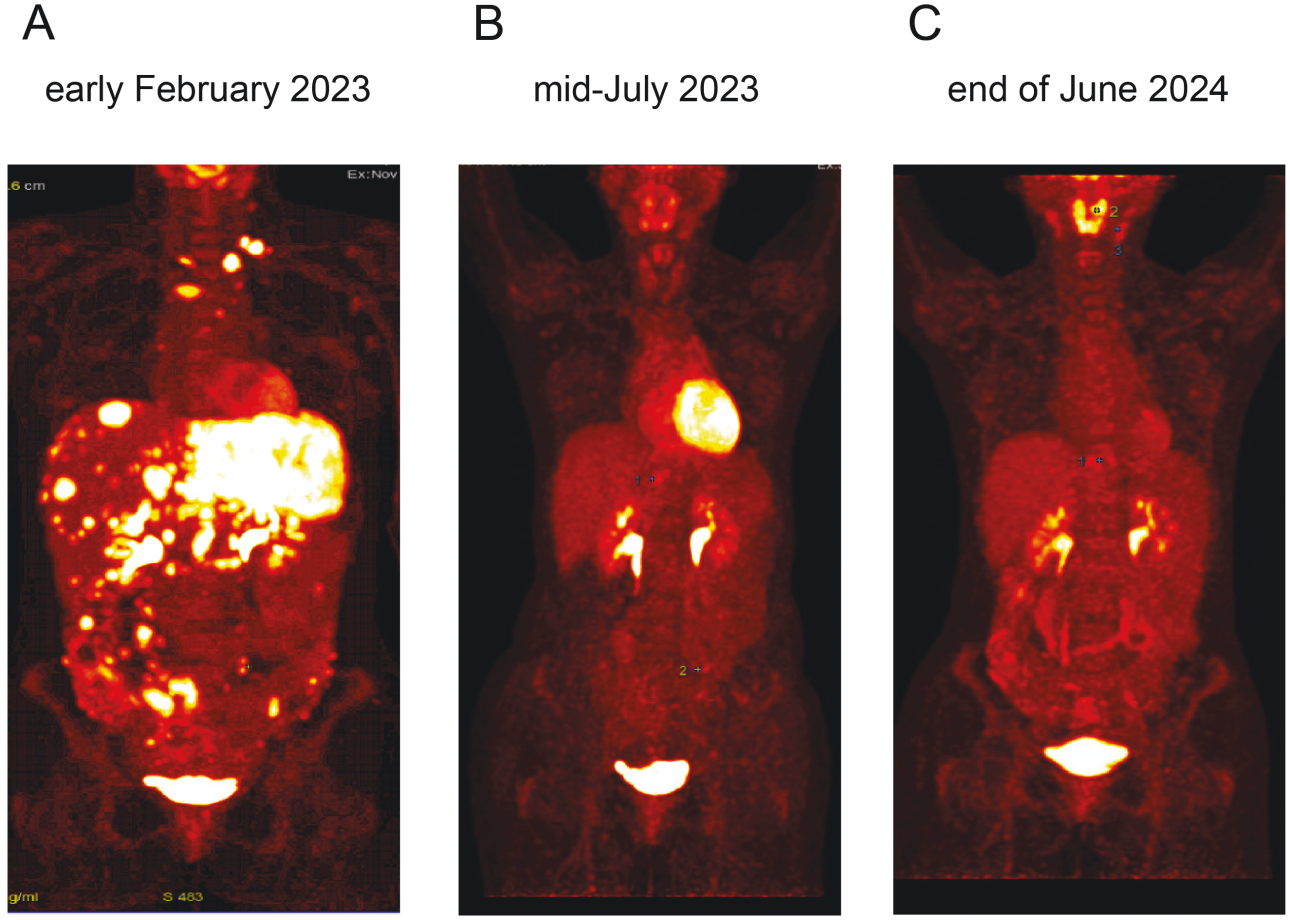
FDG–PET–CT after the failure of first-line therapy with Durvalumab/Cisplatin/Gemcitabine (A) and with a near complete response after 5 months employing Tepotinib (B) and a sustained complete response observed at a follow-up more than 16 months on treatment (C).

Following a multidisciplinary tumor board review, the patient began a palliative first-line therapy regimen consisting of Cisplatin, Gemcitabine (CisGem), and Durvalumab according to the TOPAZ-1 protocol, with concurrent antiresorptive therapy using Denosumab. From December 2022 to January 2023, she received 5 cycles of CisGem and Durvalumab, administered on a 21-day cycle (CisGem on days 1 and 8; Durvalumab on day 1). Despite initial stabilization of CA 19-9 levels (around 579.8 U/mL, normal range 2.22-37.0), tumor marker levels began to rise, reaching 1627.6 U/mL. A follow-up PET–CT scan in early February 2023 confirmed disease progression with new osseous lesions in thoracic vertebrae 6 and 11, and additional hepatic lesions (Figure 1A).

### Molecular profiling and targeted therapy

**Figure 2.**
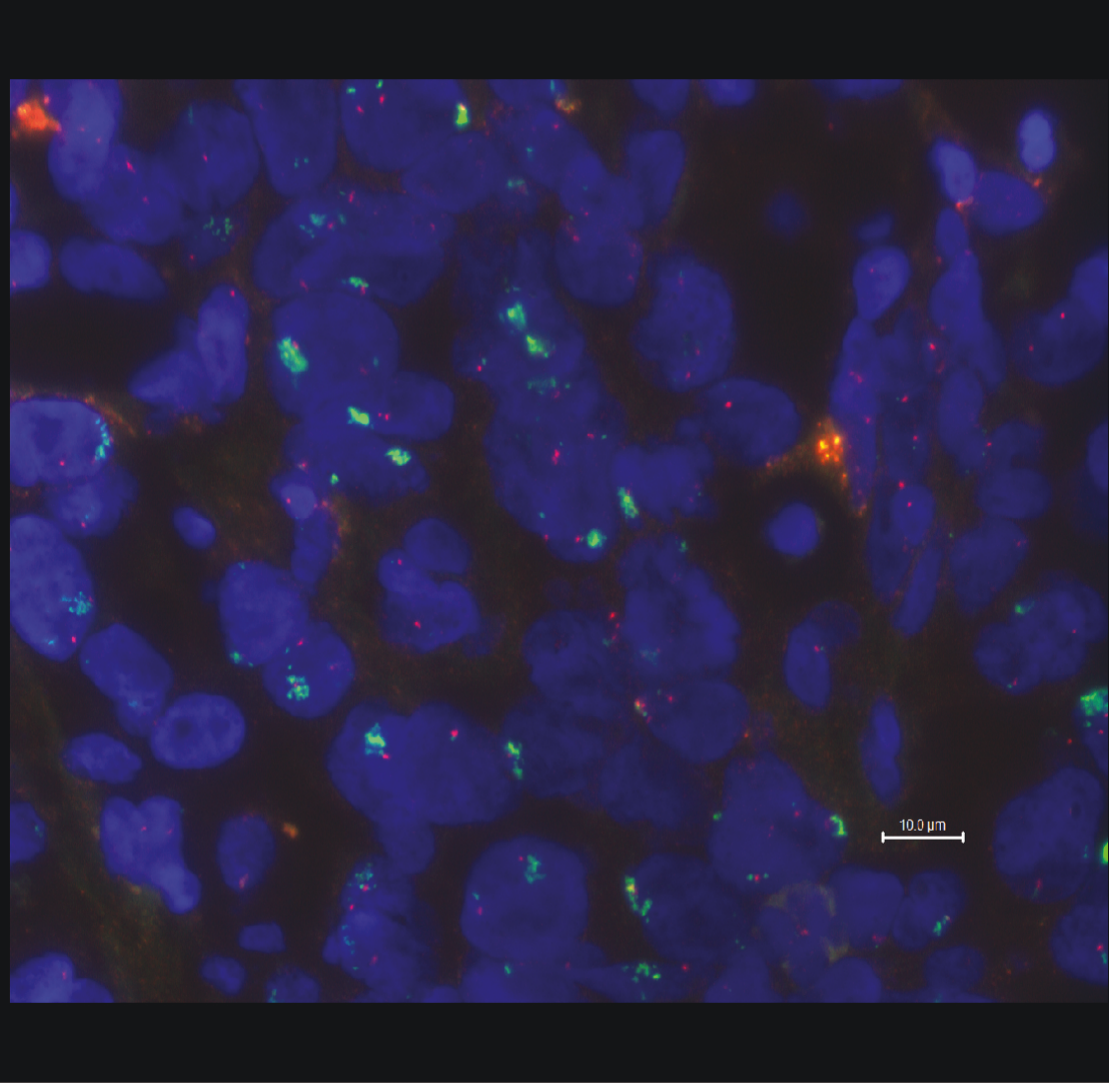
FISH analysis of interphase nuclei demonstrating strong amplified *MET* on 7q31.2 (green signal) compared to *KMT2E* as a control (red) (ratio 5-7:1). The scale bar in the bottom right indicates 10.0 µm.

In alignment with recent ESMO guidelines recommending comprehensive molecular testing for BTC patients, next-generation sequencing (NGS) using the TrueSight 170 panel (Illumina) was performed on the initial biopsy. The analysis identified a pathogenic *TP53* mutation (p.R282W; variant allele frequency 15%) and *MET* amplification with 10 copies (tumor fraction 30%). A confirmatory FISH analysis revealed a *MET*:*KMT2E* ratio of 5-7:1 (Figure 2) when *KMT2E* on 7q22 was used as a control. Based on ESCAT guidelines, which classify *MET* amplification as an ESCAT IIIA alteration, Tepotinib was chosen as the next treatment. Given the rapid progression of the disease and the patient’s young age, Tepotinib (450 mg orally once daily) was initiated in mid-February 2023.

### Response to Tepotinib

**Figure 3.**
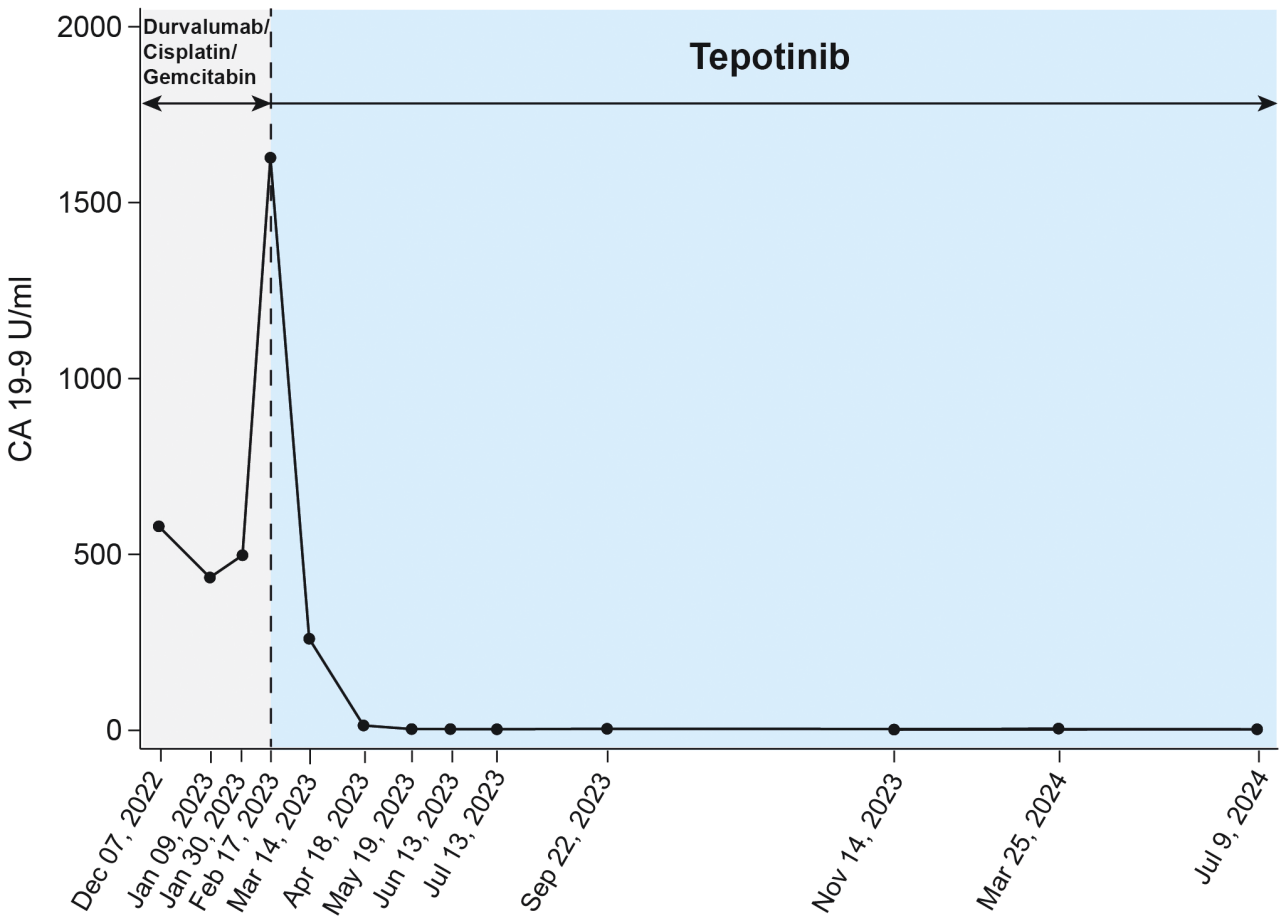
Timeline of applied systemic therapies along with Ca 19-9 levels. Ca 19-9 levels are steeply rising after 2 months of treatment with Durvalumab/Cisplatin/Gemcitabine correlating well with morphological disease progression. Tepotinib leads to a rapid, steep decline of Ca 19-9 levels which are ongoing sustained in the normal range.

Within 1 month of starting Tepotinib, a sonographic evaluation showed a reduction in the size of a large liver lesion from 12.3 cm to 11.7 cm, and CA 19-9 levels decreased significantly from 1627.6 U/mL to 260 U/mL (–84%) (Figure 3). The treatment was generally well tolerated, with only mild edema, which responded well to diuretic therapy with furosemide, and no need for dose adjustments.

After 5 months on Tepotinib, a PET–CT scan revealed a partial (near-complete) metabolic remission, and CA 19-9 levels had normalized (Figure 1B). Continued therapy over an additional 4 months led to a complete metabolic remission, which has persisted up to the preparation of this report in July 2024 (Figure 1C). The patient has experienced excellent tolerability and quality of life, with the duration of response extending up to 17 months (Figure 3).

## Discussion

Despite increasing incidences, biliary tract cancers (BTCs) remain classified as rare cancers. *MET*-amplified BTCs constitute only 2%-4% of all BTC cases, making them exceedingly rare.^9^ Consequently, evidence for treatment predominantly stems from n-of-1 trials or case reports. Here, we present a case of *MET* amplified BTC in a very young patient, treated with Tepotinib following the failure of Durvalumab, Cisplatin, and Gemcitabine therapy. To our knowledge, this is the first reported case of *MET* amplified BTC treated with Tepotinib, demonstrating an exceptional long-term response. This case underscores the efficacy of Tepotinib compared to other selective or unselective MET inhibitors (Table 1). In our case, we favored Tepotinib over Capmatinib primarily due to its once-daily dosing schedule, which is more compatible with the active lifestyle of a young patient. Whether Tepotinib’s specific pharmacokinetic characteristics—ensuring small peak-to-trough variation over 24 h—lead to better target inhibition and prolonged response duration remains speculative and is beyond the scope of this case report.^15,16^

**Table 1.** Summary of published cases of biliary tract cancers with alterations involving *MET* and targeted treatment with MET inhibitors.

Type	Alteration	Co-alterations	GCN	Drug	DOR (months)	Reference
iCC	*CAPZA2:MET*; *MET* amp	*TP53 c.991C > T* *RB1 c.2087G > C*	6.3	Capmatinib	4	^10^
iCC	*MET* amp	*TP53* mut, *TERT* mut *CDK6*, *CUL4A*, *FGF14,* and *IRS2* amp	NA	Capmatinib	6	^11^
iCC	*MET* amp	TMB-H, 11.52 Muts/Mb	NA	Savolitinib	12	^12^
GBC	*MET* amp	*TP53* mut *RB1* mut *RICTOR* amp *ATRX* mut *AR1D1A* mut	98	Capmatinib	6	^13^
iCC	*EHBP1:MET*		NA	Crizotinib	8	^14^

Abbreviations: amp, amplification; DOR, duration of response; GBC, gallbladder cancer; GCN, gene copy number; iCC, intrahepatic Cholangiocarcinoma; mut, mutation; Muts/Mb, Mutations per Megabase.

In cohort B of the Vision trial- a prospective trial evaluating Tepotinib in *MET* amplified lung cancer-, the median duration of Tepotinib treatment was 3.6 months (range, 0.1-26.8), whereas our patient exhibited an ongoing response lasting over 18 months.^17^ High-level *MET* amplification is defined using various criteria, including a *MET* ratio ≥ 2.0 or ≥2.2, or a *MET* gene copy number (GCN) ≥ 6 or ≥10, as identified by fluorescence in situ hybridization (FISH) or next-generation sequencing (NGS) on tissue biopsies.^17^ Our patient’s diagnosis aligned with the stringent criterion of *MET* GCN ≥ 10 and a *MET*: *KMT2E* ratio of over 5:1, consistent with both NGS and FISH reports. The influence of co-alterations on the efficacy of targeting MET is not well understood, particularly in BTCs.^18^ Our patient also had a co-occurring *TP53* mutation, which did not appear to impact the efficacy of Tepotinib. *MET* amplification is thought to drive overexpression of the MET receptor and its constitutive, ligand-independent activation, thereby dysregulating the MET pathway and promoting tumor growth.

This case also aligns with published characteristics of *MET*-amplified tumors. First, it supports findings from early onset cholangiocarcinoma (CCC) studies that *MET* amplified CCC is twice as common in younger patients compared to older individuals.^9^ To our knowledge, this is the youngest patient at our clinic treated for metastatic BTC. Among younger BTC patients (≤50 years), *MET* amplification ranks fourth after alterations in *FGFR2*, *BRAF*, and *IDH1*. Our patient also had a history of inflammatory bowel disease, which is sometimes associated with chronic inflammation of the biliary tracts. Although no bile duct disease was identified at presentation, we cannot exclude the possibility of an underlying precancerous condition.

Secondly, this clinical case corroborates the aggressive nature of *MET* amplified tumors, similar to observations in lung cancer from cohort B of the vision trial. Little is known about predictive genetic markers for the response or clinical efficacy of the novel first-line standard treatment with Durvalumab, Cisplatin, and Gemcitabine. Data on *MET* amplification is absent from retrospective biomarker-based analyses of the TOPAZ-1 trial or larger real-world datasets. Our clinical case suggests that *MET* amplified BTCs might present a risk for resistance to chemotherapy and checkpoint inhibitors, leading to rapid deterioration if not treated with a targeted agent as a second-line therapy. Therefore, our case addresses an unmet need in managing a difficult-to-treat molecularly defined entity and proposes a potential new standard treatment option.

## Data Availability

The data underlying this article cannot be shared publicly due to patient privacy concerns.

## References

[1] Valle JW , Kelley RK, Nervi B, Oh DY, Zhu AX. Biliary tract cancer. Lancet. 2021;397:428-444. https://doi.org/10.1016/S0140-6736(21)00153-7 33516341

[2] Izquierdo-Sanchez L , Lamarca A, Casta AL, et al. Cholangiocarcinoma landscape in Europe: diagnostic, prognostic and therapeutic insights from the ENSCCA Registry. J Hepatol. 2022;76:1109-1121. 35167909 10.1016/j.jhep.2021.12.010

[3] Oh DY , He AR, Qin S, et al. Durvalumab plus gemcitabine and cisplatin in advanced biliary tract cancer. NEJM Évid. 2022;1:EVIDoa2200015. 38319896 10.1056/EVIDoa2200015

[4] Kelley RK , Ueno M, Yoo C, et al; KEYNOTE-966 Investigators. Pembrolizumab in combination with gemcitabine and cisplatin compared with gemcitabine and cisplatin alone for patients with advanced biliary tract cancer (KEYNOTE-966): a randomised, double-blind, placebo-controlled, phase 3 trial. Lancet. 2023;401:1853-1865. https://doi.org/10.1016/S0140-6736(23)00727-4 37075781

[5] Verdaguer H , Saurí T, Acosta DA, et al. ESMO scale for clinical actionability of molecular targets driving targeted treatment in patients with cholangiocarcinoma. Clin Cancer Res. 2022;28:1662-1671. https://doi.org/10.1158/1078-0432.ccr-21-2384 35042699

[6] Doleschal B , Taghizadeh H, Webersinke G, et al. Real world evidence reveals improved survival outcomes in biliary tract cancer through molecular matched targeted treatment. Sci Rep. 2023;13:15421. https://doi.org/10.1038/s41598-023-42083-4 37723192 PMC10507096

[7] Zhang D , Dorman K, Heinrich K, et al. A retrospective analysis of biliary tract cancer patients presented to the molecular tumor board at the comprehensive cancer center Munich. Target Oncol. 2023;18:767-776. https://doi.org/10.1007/s11523-023-00985-3 37594677 PMC10517894

[8] Paik PK , Felip E, Veillon R, et al. Tepotinib in non–small-cell lung cancer with MET Exon 14 skipping mutations. N Engl J Med. 2020;383:931-943. https://doi.org/10.1056/NEJMoa2004407 32469185 PMC8422679

[9] Pappas L , Baiev I, Reyes S, et al. The Cholangiocarcinoma in the Young (CITY) study: tumor biology, treatment patterns, and survival outcomes in adolescent young adults with cholangiocarcinoma. JCO Precis Oncol. 2023;7:e2200594. https://doi.org/10.1200/PO.22.00594 37561981 PMC10581631

[10] Turpin A , Descarpentries C, Grégoire V, et al. Response to Capmatinib in a MET fusion-positive Cholangiocarcinoma. Oncologist. 2023;28:80-83. https://doi.org/10.1093/oncolo/oyac194 36434677 PMC9847551

[11] Lefler DS , Tierno MB, Bashir B. Partial treatment response to capmatinib in MET-amplified metastatic intrahepatic cholangiocarcinoma: case report & review of literature. Cancer Biol Ther. 2022;23:112-116. https://doi.org/10.1080/15384047.2022.2029128 35129063 PMC8820818

[12] Zhou K , Liu Y, Zhu H. Dramatic response and acquired resistance to savolitinib in advanced intrahepatic cholangiocarcinoma with MET amplification: a case report and literature review. Front Oncol. 2023;13:1254026. https://doi.org/10.3389/fonc.2023.1254026 38023194 PMC10652553

[13] Yamamura S , Kanai M, Takeuchi Y, et al. Response to capmatinib in a patient with neuroendocrine carcinoma of the gallbladder origin harboring MET amplification. Int Cancer Conf J. 2024;13:83-87. https://doi.org/10.1007/s13691-023-00643-5 38524646 PMC10957854

[14] Yu Y , Liu Q, Li W, et al. Identification of a Novel EHBP1‐MET fusion in an intrahepatic cholangiocarcinoma responding to crizotinib. The Oncologist. 2020;25:1005-1008. 32897609 10.1634/theoncologist.2020-0535 PMC7938406

[15] Mathieu LN , Larkins E, Akinboro O, et al. FDA approval summary: capmatinib and tepotinib for the treatment of metastatic NSCLC Harboring MET Exon 14 skipping mutations or alterations. Clin Cancer Res. 2021;28:249-254. https://doi.org/10.1158/1078-0432.ccr-21-1566 34344795

[16] Falchook GS , Kurzrock R, Amin HM, et al. First-in-man phase I Trial of the selective MET inhibitor tepotinib in patients with advanced solid tumors. Clin Cancer Res. 2020;26:1237-1246. https://doi.org/10.1158/1078-0432.CCR-19-2860 31822497

[17] Le X , Paz-Ares LG, Meerbeeck JV, et al. Tepotinib in patients with non–small cell lung cancer with high-level MET amplification detected by liquid biopsy: VISION Cohort B. Cell Rep Med. 2023;4:101280. 10.1016/j.xcrm.2023.101280 PMC10694660 37944528

[18] Kendre G , Murugesan K, Brummer T, et al. Charting co-mutation patterns associated with actionable drivers in intrahepatic cholangiocarcinoma. J Hepatol. 2023;78:614-626. https://doi.org/10.1016/j.jhep.2022.11.030 36528236

